# 2-Hy­droxy­ethanaminium 2,4-dinitro­phenolate hemihydrate

**DOI:** 10.1107/S1600536810031983

**Published:** 2010-08-18

**Authors:** Yong Yan, Guoxia Han

**Affiliations:** a School of Chemistry and Chemical Engineering, Nanjing University, Nanjing 210093, People’s Republic of China

## Abstract

In the title salt, C_2_H_8_NO^+^·C_6_H_3_N_2_O_5_^−^·0.5H_2_O, the anions, cations and water mol­ecules are linked *via* N—H⋯O and O—H⋯O hydrogen bonds, forming a three-dimensionl network.

## Related literature

For comparable structures, see: Goddard *et al.* (2002 ▶); Iwasaki & Kawano (1977 ▶); Kunnert *et al.* (1995 ▶); Rais & Bergman (2004 ▶); Sieler *et al.* (1994 ▶); Yuan *et al.* (2005 ▶).
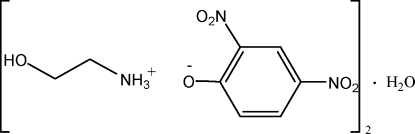


## Experimental

### 

#### Crystal data

C_2_H_8_NO^+^·C_6_H_3_N_2_O_5_^−^·0.5H_2_O
*M* *_r_* = 254.20
Orthorhombic, 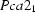

*a* = 24.5688 (4) Å
*b* = 10.5945 (2) Å
*c* = 8.4238 (2) Å
*V* = 2192.67 (8) Å^3^
*Z* = 8
Mo *K*α radiation
μ = 0.14 mm^−1^
*T* = 153 K
0.56 × 0.45 × 0.33 mm

#### Data collection

Rigaku R-AXIS RAPID diffractometer
20722 measured reflections
2686 independent reflections
2646 reflections with *I* > 2σ(*I*)
*R*_int_ = 0.018

#### Refinement

*R*[*F* ^2^ > 2σ(*F* ^2^)] = 0.024
*wR*(*F* ^2^) = 0.066
*S* = 1.00
2686 reflections
357 parameters
1 restraint
H atoms treated by a mixture of independent and constrained refinement
Δρ_max_ = 0.25 e Å^−3^
Δρ_min_ = −0.17 e Å^−3^

### 

Data collection: *RAPID-AUTO* (Rigaku/MSC, 2004 ▶); cell refinement: *RAPID-AUTO*; data reduction: *RAPID-AUTO*; program(s) used to solve structure: *SHELXS97* (Sheldrick, 2008 ▶); program(s) used to refine structure: *SHELXL97* (Sheldrick, 2008 ▶); molecular graphics: *SHELXTL* (Sheldrick, 2008 ▶); software used to prepare material for publication: *SHELXTL*.

## Supplementary Material

Crystal structure: contains datablocks global, I. DOI: 10.1107/S1600536810031983/bt5319sup1.cif

Structure factors: contains datablocks I. DOI: 10.1107/S1600536810031983/bt5319Isup2.hkl

Additional supplementary materials:  crystallographic information; 3D view; checkCIF report

## Figures and Tables

**Table 1:** Hydrogen-bond geometry (Å, °)

*D*—H⋯*A*	*D*—H	H⋯*A*	*D*⋯*A*	*D*—H⋯*A*
O11—H11*O*⋯O1	0.84 (3)	1.76 (3)	2.5914 (14)	168 (3)
O12—H12*O*⋯O6	0.77 (3)	1.94 (3)	2.6963 (16)	166 (3)
O13—H0*A*⋯O1	0.84 (2)	1.94 (2)	2.7315 (13)	156 (2)
O13—H0*A*⋯O5	0.84 (2)	2.31 (2)	2.8938 (15)	127 (2)
O13—H0*B*⋯O12^i^	0.85 (2)	1.97 (2)	2.8214 (15)	171 (2)
N5—H5*B*⋯O9^ii^	0.92 (2)	2.13 (2)	2.9620 (15)	150 (2)
N5—H5*C*⋯O11^iii^	0.92 (2)	1.85 (2)	2.7620 (16)	171 (2)
N5—H5*A*⋯O13	0.82 (2)	2.11 (2)	2.9038 (14)	161 (2)
N6—H6*C*⋯O5^ii^	0.91 (2)	2.07 (2)	2.9127 (16)	155 (2)
N6—H6*B*⋯O6^ii^	0.87 (3)	1.93 (3)	2.7453 (17)	155 (2)
N6—H6*B*⋯O10^ii^	0.87 (3)	2.34 (2)	2.9517 (16)	127 (2)
N6—H6*A*⋯O13	0.93 (2)	1.87 (2)	2.7937 (17)	175 (2)

Symmetry codes: (i) 
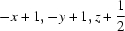
; (ii) 
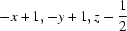
; (iii) 
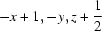
.

**Fig. 1.**
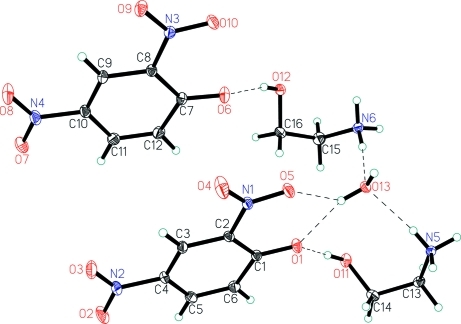
The cell unit of (I) with atom labels, showing 40% probability displacement ellipsoids. Hydrogen bonds are illustrated as thin lines.

**Fig. 2.**
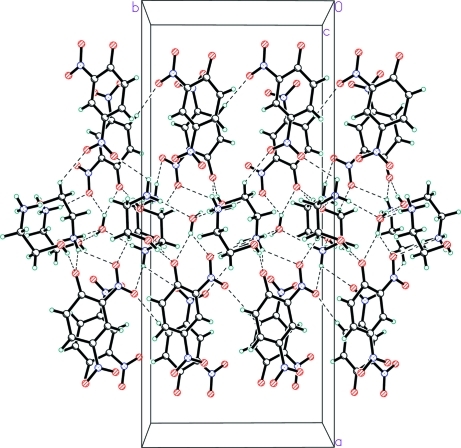
A diagram of crystal packing viewed down along the b axis. Hydrogen bonds are drawn as dashed lines.

## supplementary crystallographic information

### Comment

As shown in Fig. 1, compound (I) consists of two crystallographically independent ionic O^+^—H···O^-^ hydrogen bonding (Table 2) pairs of ethanolammonium DNPL (A and B for containing O1 and O6, respectively), and one water molecule. The ion pairs of A and B are asociated *via* the water molecule by a N—H···O hydrogen bonds (Table 2).

Following analysis shows the the feature of quinoic-phenolic resonance of DNPL in ionic pairs A. The C1—C6 and C1—C2 bond lengths are extremely longer than the normal length (1.38 Å), and the C3—C4 bond length is abviously longer than the normal length. The C2—C3 bond length is the shortest one in DNPL, and is slightly shorter than the normal one (Table 1). The N1—C6 [1.4416 (16) Å] and N2—C4 [1.4478 (17) Å] bond lengths are obviously shorter than that observed in 2,4-dinitrophenol [1.484 (5) Å, Iwasaki & Kawano, 1977]. The C1—O1 bond length [1.2671 (16) Å] is shorter than that of phenolates, 1.317 (4)Å for a naphtholate (Yuan *et al.*, 2005), 1.283 (2)–1.331 (6)Å for phenolates in different compounds (Sieler *et al.*, 1994; Kunnert *et al.*, 1995; Goddard *et al.*, 2002; Rais *et al.*, 2004).

The nitro groups of the DNPL at C2 and C4 are twisted relatively to the aromatic ring with dihedral angles lesser than about 4°, which are indicated by torsion angles in Table 2. This twist of the nitro group relative to the benzene ring is a result of the participation of nitro O atoms in O—H···O hydrogen bonds (Table 2).

The N—H···O and O—H···O hydrogen bonding in the structure leads to a three-dimensional hydrogen-bonded netwerk (Table 2). As shown in Fig. 2, the water molecule acts as well a hydrogen-bond donor for DNPL anion and ethanolammonium cation. Furthermore, the DNPL anions are linked to ethanolammonium cations *via* various hydrogen bonds of N—H···O (Table 2 & Fig. 2).

### Experimental

Admixture of ethanolamine, DNP and water in the molar ratio of 2:1:1 were heated to a temperature where clear solutions resulted. The cystals of (I) were formed by making the resulting solutions standing overnight at 293k.

### Refinement

The H atoms bonded to N and O were taken from a difference fourier map, and were refined. Others were placed in calculated positions and allowed to ride on their parent atoms at distances of 0.95 (phenyl), 0.99 (methylene), with *U*_iso_(H) values 1.2 times *U*_eq_ of the parent atoms.

### Crystal data

**Table:**

C_2_H_8_NO^+^·C_6_H_3_N_2_O_5_^−^·0.5H_2_O	*F*(000) = 1064
*M**_r_* = 254.20	*D*_x_ = 1.540 Mg m^−^^3^
Orthorhombic, *P**c**a*2_1_	Mo *K*α radiation, λ = 0.71073 Å
Hall symbol: P 2c -2ac	Cell parameters from 20146 reflections
*a* = 24.5688 (4) Å	θ = 3.1–27.5°
*b* = 10.5945 (2) Å	µ = 0.14 mm^−^^1^
*c* = 8.4238 (2) Å	*T* = 153 K
*V* = 2192.67 (8) Å^3^	Prism, yellow
*Z* = 8	0.56 × 0.45 × 0.33 mm

### Data collection

**Table:**

Rigaku R-AXIS RAPID diffractometer	2646 reflections with *I* > 2σ(*I*)
Radiation source: Rotating Anode	*R*_int_ = 0.018
graphite	θ_max_ = 27.5°, θ_min_ = 3.1°
ω scans	*h* = −31→31
20722 measured reflections	*k* = −13→13
2686 independent reflections	*l* = −10→10

### Refinement

**Table:**

Refinement on *F*^2^	Secondary atom site location: difference Fourier map
Least-squares matrix: full	Hydrogen site location: inferred from neighbouring sites
*R*[*F*^2^ > 2σ(*F*^2^)] = 0.024	H atoms treated by a mixture of independent and constrained refinement
*wR*(*F*^2^) = 0.066	*w* = 1/[σ^2^(*F*_o_^2^) + (0.0506*P*)^2^ + 0.256*P*] where *P* = (*F*_o_^2^ + 2*F*_c_^2^)/3
*S* = 1.00	(Δ/σ)_max_ = 0.001
2686 reflections	Δρ_max_ = 0.25 e Å^−^^3^
357 parameters	Δρ_min_ = −0.17 e Å^−^^3^
1 restraint	Extinction correction: *SHELXL97* (Sheldrick, 2008), Fc^*^=kFc[1+0.001xFc^2^λ^3^/sin(2θ)]^-1/4^
Primary atom site location: structure-invariant direct methods	Extinction coefficient: 0.0213 (13)

### Special details

**Table:**

Geometry. All e.s.d.'s (except the e.s.d. in the dihedral angle between two l.s. planes) are estimated using the full covariance matrix. The cell e.s.d.'s are taken into account individually in the estimation of e.s.d.'s in distances, angles and torsion angles; correlations between e.s.d.'s in cell parameters are only used when they are defined by crystal symmetry. An approximate (isotropic) treatment of cell e.s.d.'s is used for estimating e.s.d.'s involving l.s. planes.
Refinement. Refinement of *F*^2^ against ALL reflections. The weighted *R*-factor *wR* and goodness of fit *S* are based on *F*^2^, conventional *R*-factors *R* are based on *F*, with *F* set to zero for negative *F*^2^. The threshold expression of *F*^2^ > σ(*F*^2^) is used only for calculating *R*-factors(gt) *etc*. and is not relevant to the choice of reflections for refinement. *R*-factors based on *F*^2^ are statistically about twice as large as those based on *F*, and *R*- factors based on ALL data will be even larger.

### Fractional atomic coordinates and isotropic or equivalent isotropic displacement parameters (Å^2^)

**Table:**

	*x*	*y*	*z*	*U*_iso_*/*U*_eq_	
O1	0.40970 (4)	0.16257 (9)	0.70708 (14)	0.0246 (2)	
O2	0.15648 (4)	0.16118 (13)	0.7433 (2)	0.0436 (4)	
O3	0.17398 (5)	0.31833 (12)	0.89817 (19)	0.0390 (3)	
O4	0.35318 (5)	0.43069 (14)	1.0234 (2)	0.0489 (4)	
O5	0.42268 (4)	0.35022 (10)	0.91162 (16)	0.0300 (3)	
O6	0.38672 (4)	0.63686 (10)	0.71654 (14)	0.0262 (2)	
O7	0.13452 (4)	0.69048 (12)	0.70476 (19)	0.0384 (3)	
O8	0.15347 (5)	0.83799 (13)	0.87468 (18)	0.0407 (3)	
O9	0.33436 (4)	0.93189 (10)	1.00421 (14)	0.0292 (2)	
O10	0.40336 (4)	0.83871 (10)	0.89816 (14)	0.0265 (2)	
O11	0.46306 (4)	0.03331 (10)	0.49735 (13)	0.0236 (2)	
O12	0.45349 (4)	0.58299 (10)	0.47171 (13)	0.0220 (2)	
N1	0.37306 (4)	0.35618 (10)	0.92905 (16)	0.0211 (2)	
N2	0.18870 (5)	0.23265 (12)	0.81025 (19)	0.0276 (3)	
N3	0.35365 (5)	0.85094 (10)	0.91370 (14)	0.0194 (2)	
N4	0.16743 (5)	0.75211 (13)	0.78413 (18)	0.0270 (3)	
N5	0.56485 (5)	0.01705 (10)	0.68611 (15)	0.0185 (2)	
N6	0.53325 (5)	0.39558 (11)	0.44028 (16)	0.0218 (2)	
C1	0.35954 (5)	0.17880 (12)	0.73496 (16)	0.0175 (2)	
C2	0.31902 (6)	0.09935 (12)	0.66134 (18)	0.0224 (3)	
H2	0.3308	0.0330	0.5938	0.027*	
C3	0.26441 (6)	0.11540 (13)	0.68459 (19)	0.0236 (3)	
H3	0.2390	0.0615	0.6335	0.028*	
C4	0.24637 (6)	0.21292 (11)	0.78519 (17)	0.0210 (3)	
C5	0.28234 (5)	0.29052 (12)	0.86297 (18)	0.0202 (3)	
H5	0.2694	0.3552	0.9314	0.024*	
C6	0.33790 (5)	0.27319 (12)	0.84031 (16)	0.0176 (3)	
C7	0.33707 (5)	0.66684 (12)	0.73307 (15)	0.0186 (3)	
C8	0.29498 (6)	0.59359 (13)	0.65700 (19)	0.0243 (3)	
H8	0.3054	0.5241	0.5926	0.029*	
C9	0.24074 (6)	0.61971 (12)	0.67346 (19)	0.0249 (3)	
H9	0.2143	0.5687	0.6220	0.030*	
C10	0.22461 (5)	0.72307 (13)	0.76756 (18)	0.0217 (3)	
C11	0.26181 (5)	0.79730 (12)	0.84427 (16)	0.0194 (3)	
H11	0.2502	0.8663	0.9078	0.023*	
C12	0.31712 (5)	0.77025 (12)	0.82799 (16)	0.0180 (3)	
C13	0.54204 (6)	−0.08272 (12)	0.58103 (18)	0.0209 (3)	
H13A	0.5540	−0.1666	0.6191	0.025*	
H13B	0.5561	−0.0711	0.4718	0.025*	
C14	0.48060 (6)	−0.07767 (13)	0.57902 (19)	0.0233 (3)	
H14A	0.4660	−0.1535	0.5251	0.028*	
H14B	0.4666	−0.0767	0.6892	0.028*	
C15	0.48229 (6)	0.39280 (14)	0.34614 (19)	0.0273 (3)	
H15A	0.4720	0.3040	0.3251	0.033*	
H15B	0.4886	0.4346	0.2427	0.033*	
C16	0.43622 (5)	0.45829 (13)	0.4314 (2)	0.0246 (3)	
H16A	0.4037	0.4621	0.3621	0.029*	
H16B	0.4265	0.4110	0.5288	0.029*	
O13	0.51234 (4)	0.26120 (9)	0.71903 (13)	0.0197 (2)	
H0A	0.4788 (9)	0.249 (2)	0.732 (3)	0.036 (5)*	
H0B	0.5255 (8)	0.3027 (19)	0.797 (3)	0.029 (5)*	
H5A	0.5523 (9)	0.088 (2)	0.673 (3)	0.037 (6)*	
H5B	0.6013 (9)	0.0271 (17)	0.667 (3)	0.031 (5)*	
H5C	0.5571 (8)	−0.0081 (19)	0.788 (3)	0.028 (5)*	
H6A	0.5286 (8)	0.3495 (18)	0.533 (3)	0.024 (4)*	
H6B	0.5606 (10)	0.366 (2)	0.386 (3)	0.041 (6)*	
H6C	0.5420 (8)	0.4771 (19)	0.461 (3)	0.028 (5)*	
H11O	0.4426 (10)	0.078 (2)	0.555 (4)	0.042 (6)*	
H12O	0.4350 (10)	0.610 (2)	0.537 (4)	0.044 (7)*	

### Atomic displacement parameters (Å^2^)

**Table:**

	*U*^11^	*U*^22^	*U*^33^	*U*^12^	*U*^13^	*U*^23^
O1	0.0164 (4)	0.0285 (5)	0.0289 (5)	0.0015 (3)	0.0016 (4)	−0.0118 (4)
O2	0.0166 (5)	0.0491 (7)	0.0652 (10)	−0.0100 (5)	−0.0046 (6)	−0.0124 (7)
O3	0.0200 (5)	0.0422 (6)	0.0548 (8)	0.0079 (4)	0.0030 (5)	−0.0084 (6)
O4	0.0238 (5)	0.0577 (8)	0.0653 (9)	0.0005 (5)	0.0029 (6)	−0.0452 (8)
O5	0.0154 (4)	0.0274 (5)	0.0472 (7)	−0.0029 (4)	0.0007 (5)	−0.0153 (5)
O6	0.0180 (4)	0.0364 (5)	0.0242 (5)	0.0073 (4)	−0.0034 (4)	−0.0102 (5)
O7	0.0173 (5)	0.0440 (6)	0.0539 (8)	−0.0048 (4)	−0.0075 (5)	−0.0008 (7)
O8	0.0223 (5)	0.0569 (7)	0.0429 (7)	0.0128 (5)	0.0000 (5)	−0.0094 (7)
O9	0.0240 (5)	0.0303 (5)	0.0333 (6)	0.0067 (4)	−0.0053 (5)	−0.0156 (5)
O10	0.0158 (4)	0.0313 (5)	0.0326 (6)	−0.0033 (4)	0.0014 (4)	−0.0080 (5)
O11	0.0221 (4)	0.0288 (5)	0.0199 (5)	0.0050 (4)	0.0004 (4)	−0.0066 (4)
O12	0.0188 (4)	0.0221 (5)	0.0250 (5)	−0.0002 (4)	0.0048 (4)	−0.0013 (4)
N1	0.0164 (5)	0.0197 (5)	0.0274 (6)	0.0004 (4)	0.0000 (5)	−0.0073 (5)
N2	0.0151 (5)	0.0309 (6)	0.0368 (7)	−0.0006 (4)	−0.0012 (5)	−0.0004 (6)
N3	0.0184 (5)	0.0197 (5)	0.0199 (6)	0.0016 (4)	−0.0014 (5)	−0.0019 (4)
N4	0.0160 (5)	0.0330 (6)	0.0319 (7)	0.0020 (5)	−0.0023 (5)	0.0046 (5)
N5	0.0168 (5)	0.0181 (5)	0.0206 (6)	0.0016 (4)	0.0013 (4)	0.0027 (4)
N6	0.0210 (5)	0.0191 (5)	0.0253 (6)	0.0023 (4)	0.0065 (5)	0.0015 (5)
C1	0.0167 (5)	0.0178 (5)	0.0178 (6)	0.0003 (4)	0.0000 (5)	−0.0013 (5)
C2	0.0221 (6)	0.0198 (6)	0.0253 (7)	−0.0014 (5)	−0.0010 (5)	−0.0060 (5)
C3	0.0206 (6)	0.0226 (6)	0.0275 (7)	−0.0052 (5)	−0.0036 (6)	−0.0027 (6)
C4	0.0129 (5)	0.0231 (5)	0.0269 (7)	−0.0009 (5)	0.0001 (5)	0.0014 (5)
C5	0.0159 (6)	0.0204 (5)	0.0242 (7)	0.0016 (4)	0.0007 (5)	−0.0024 (5)
C6	0.0155 (5)	0.0173 (5)	0.0201 (6)	−0.0015 (4)	0.0000 (5)	−0.0028 (5)
C7	0.0182 (6)	0.0218 (6)	0.0160 (6)	0.0028 (5)	−0.0025 (5)	−0.0009 (5)
C8	0.0242 (7)	0.0217 (6)	0.0269 (7)	0.0024 (5)	−0.0064 (6)	−0.0065 (5)
C9	0.0223 (7)	0.0219 (5)	0.0304 (8)	−0.0016 (5)	−0.0080 (6)	−0.0019 (6)
C10	0.0155 (6)	0.0250 (6)	0.0245 (7)	0.0014 (5)	−0.0021 (5)	0.0032 (5)
C11	0.0167 (6)	0.0221 (5)	0.0195 (6)	0.0037 (4)	−0.0009 (5)	0.0001 (5)
C12	0.0162 (6)	0.0197 (5)	0.0179 (6)	0.0008 (4)	−0.0018 (5)	−0.0012 (5)
C13	0.0242 (6)	0.0182 (5)	0.0203 (6)	0.0026 (5)	0.0045 (5)	−0.0009 (5)
C14	0.0230 (6)	0.0238 (6)	0.0231 (7)	−0.0047 (5)	0.0040 (5)	−0.0030 (6)
C15	0.0287 (7)	0.0279 (6)	0.0253 (7)	0.0012 (5)	0.0016 (6)	−0.0055 (6)
C16	0.0187 (6)	0.0240 (6)	0.0310 (8)	−0.0036 (5)	0.0015 (5)	−0.0012 (6)
O13	0.0156 (4)	0.0214 (4)	0.0220 (5)	−0.0015 (3)	0.0009 (4)	−0.0011 (4)

### Geometric parameters (Å, °)

**Table:**

O1—C1	1.2661 (16)	C2—C3	1.3665 (19)
O2—N2	1.2322 (18)	C2—H2	0.9500
O3—N2	1.2261 (19)	C3—C4	1.408 (2)
O4—N1	1.2223 (17)	C3—H3	0.9500
O5—N1	1.2293 (15)	C4—C5	1.3734 (19)
O6—C7	1.2683 (16)	C5—C6	1.3905 (17)
O7—N4	1.2357 (19)	C5—H5	0.9500
O8—N4	1.236 (2)	C7—C12	1.4421 (18)
O9—N3	1.2417 (15)	C7—C8	1.4429 (18)
O10—N3	1.2351 (15)	C8—C9	1.3680 (19)
O11—C14	1.4288 (17)	C8—H8	0.9500
O11—H11O	0.84 (3)	C9—C10	1.409 (2)
O12—C16	1.4285 (17)	C9—H9	0.9500
O12—H12O	0.77 (3)	C10—C11	1.3681 (18)
N1—C6	1.4416 (16)	C11—C12	1.3955 (17)
N2—C4	1.4478 (17)	C11—H11	0.9500
N3—C12	1.4345 (17)	C13—C14	1.5105 (19)
N4—C10	1.4449 (16)	C13—H13A	0.9900
N5—C13	1.4882 (18)	C13—H13B	0.9900
N5—H5A	0.82 (2)	C14—H14A	0.9900
N5—H5B	0.92 (2)	C14—H14B	0.9900
N5—H5C	0.92 (2)	C15—C16	1.510 (2)
N6—C15	1.4824 (19)	C15—H15A	0.9900
N6—H6A	0.93 (2)	C15—H15B	0.9900
N6—H6B	0.87 (3)	C16—H16A	0.9900
N6—H6C	0.91 (2)	C16—H16B	0.9900
C1—C6	1.4388 (18)	O13—H0A	0.84 (2)
C1—C2	1.4438 (18)	O13—H0B	0.85 (2)
			
C14—O11—H11O	111.2 (18)	O6—C7—C12	125.31 (12)
C16—O12—H12O	109.9 (18)	O6—C7—C8	120.39 (12)
O4—N1—O5	120.48 (12)	C12—C7—C8	114.28 (11)
O4—N1—C6	119.45 (11)	C9—C8—C7	122.97 (13)
O5—N1—C6	120.06 (11)	C9—C8—H8	118.5
O3—N2—O2	122.82 (13)	C7—C8—H8	118.5
O3—N2—C4	118.92 (12)	C8—C9—C10	119.23 (12)
O2—N2—C4	118.25 (13)	C8—C9—H9	120.4
O10—N3—O9	121.00 (11)	C10—C9—H9	120.4
O10—N3—C12	120.19 (11)	C11—C10—C9	121.64 (12)
O9—N3—C12	118.81 (11)	C11—C10—N4	118.79 (12)
O7—N4—O8	122.78 (13)	C9—C10—N4	119.57 (13)
O7—N4—C10	118.13 (13)	C10—C11—C12	119.09 (12)
O8—N4—C10	119.08 (13)	C10—C11—H11	120.5
C13—N5—H5A	115.5 (17)	C12—C11—H11	120.5
C13—N5—H5B	110.1 (13)	C11—C12—N3	115.94 (12)
H5A—N5—H5B	103.6 (19)	C11—C12—C7	122.78 (12)
C13—N5—H5C	105.7 (13)	N3—C12—C7	121.28 (11)
H5A—N5—H5C	108 (2)	N5—C13—C14	110.97 (11)
H5B—N5—H5C	113.8 (18)	N5—C13—H13A	109.4
C15—N6—H6A	109.6 (12)	C14—C13—H13A	109.4
C15—N6—H6B	111.3 (16)	N5—C13—H13B	109.4
H6A—N6—H6B	110 (2)	C14—C13—H13B	109.4
C15—N6—H6C	108.8 (13)	H13A—C13—H13B	108.0
H6A—N6—H6C	111.6 (19)	O11—C14—C13	109.63 (11)
H6B—N6—H6C	105 (2)	O11—C14—H14A	109.7
O1—C1—C6	124.65 (12)	C13—C14—H14A	109.7
O1—C1—C2	120.82 (12)	O11—C14—H14B	109.7
C6—C1—C2	114.53 (11)	C13—C14—H14B	109.7
C3—C2—C1	122.89 (12)	H14A—C14—H14B	108.2
C3—C2—H2	118.6	N6—C15—C16	111.68 (13)
C1—C2—H2	118.6	N6—C15—H15A	109.3
C2—C3—C4	119.12 (12)	C16—C15—H15A	109.3
C2—C3—H3	120.4	N6—C15—H15B	109.3
C4—C3—H3	120.4	C16—C15—H15B	109.3
C5—C4—C3	121.60 (12)	H15A—C15—H15B	107.9
C5—C4—N2	118.28 (12)	O12—C16—C15	108.38 (11)
C3—C4—N2	120.12 (12)	O12—C16—H16A	110.0
C4—C5—C6	119.15 (12)	C15—C16—H16A	110.0
C4—C5—H5	120.4	O12—C16—H16B	110.0
C6—C5—H5	120.4	C15—C16—H16B	110.0
C5—C6—C1	122.63 (12)	H16A—C16—H16B	108.4
C5—C6—N1	115.89 (11)	H0A—O13—H0B	111 (2)
C1—C6—N1	121.48 (11)		
			
O1—C1—C2—C3	−178.24 (14)	C12—C7—C8—C9	−0.1 (2)
C6—C1—C2—C3	2.5 (2)	C7—C8—C9—C10	0.5 (2)
C1—C2—C3—C4	−0.4 (2)	C8—C9—C10—C11	−0.7 (2)
C2—C3—C4—C5	−1.4 (2)	C8—C9—C10—N4	179.33 (14)
C2—C3—C4—N2	179.31 (13)	O7—N4—C10—C11	174.57 (14)
O3—N2—C4—C5	0.8 (2)	O8—N4—C10—C11	−4.5 (2)
O2—N2—C4—C5	−178.37 (15)	O7—N4—C10—C9	−5.4 (2)
O3—N2—C4—C3	−179.87 (15)	O8—N4—C10—C9	175.54 (14)
O2—N2—C4—C3	1.0 (2)	C9—C10—C11—C12	0.4 (2)
C3—C4—C5—C6	0.7 (2)	N4—C10—C11—C12	−179.61 (12)
N2—C4—C5—C6	−179.92 (13)	C10—C11—C12—N3	−179.11 (12)
C4—C5—C6—C1	1.6 (2)	C10—C11—C12—C7	0.1 (2)
C4—C5—C6—N1	−178.49 (12)	O10—N3—C12—C11	−175.46 (12)
O1—C1—C6—C5	177.64 (13)	O9—N3—C12—C11	5.19 (18)
C2—C1—C6—C5	−3.1 (2)	O10—N3—C12—C7	5.35 (19)
O1—C1—C6—N1	−2.2 (2)	O9—N3—C12—C7	−174.00 (13)
C2—C1—C6—N1	177.00 (12)	O6—C7—C12—C11	−178.70 (13)
O4—N1—C6—C5	3.9 (2)	C8—C7—C12—C11	−0.2 (2)
O5—N1—C6—C5	−177.39 (13)	O6—C7—C12—N3	0.4 (2)
O4—N1—C6—C1	−176.23 (15)	C8—C7—C12—N3	178.91 (12)
O5—N1—C6—C1	2.5 (2)	N5—C13—C14—O11	69.73 (14)
O6—C7—C8—C9	178.49 (14)	N6—C15—C16—O12	54.43 (16)

### Hydrogen-bond geometry (Å, °)

**Table:**

*D*—H···*A*	*D*—H	H···*A*	*D*···*A*	*D*—H···*A*
O11—H11O···O1	0.84 (3)	1.76 (3)	2.5914 (14)	168 (3)
O12—H12O···O6	0.77 (3)	1.94 (3)	2.6963 (16)	166 (3)
O13—H0A···O1	0.84 (2)	1.94 (2)	2.7315 (13)	156 (2)
O13—H0A···O5	0.84 (2)	2.31 (2)	2.8938 (15)	127 (2)
O13—H0B···O12^i^	0.85 (2)	1.97 (2)	2.8214 (15)	171 (2)
N5—H5B···O9^ii^	0.92 (2)	2.13 (2)	2.9620 (15)	150 (2)
N5—H5C···O11^iii^	0.92 (2)	1.85 (2)	2.7620 (16)	171 (2)
N5—H5A···O13	0.82 (2)	2.11 (2)	2.9038 (14)	161 (2)
N6—H6C···O5^ii^	0.91 (2)	2.07 (2)	2.9127 (16)	155 (2)
N6—H6B···O6^ii^	0.87 (3)	1.93 (3)	2.7453 (17)	155 (2)
N6—H6B···O10^ii^	0.87 (3)	2.34 (2)	2.9517 (16)	127 (2)
N6—H6A···O13	0.93 (2)	1.87 (2)	2.7937 (17)	175 (2)

Symmetry codes: (i) −*x*+1, −*y*+1, *z*+1/2; (ii) −*x*+1, −*y*+1, *z*−1/2; (iii) −*x*+1, −*y*, *z*+1/2.
